# Preparing for the Nordic Skiing Events at the Beijing Olympics in 2022: Evidence-Based Recommendations and Unanswered Questions

**DOI:** 10.1007/s42978-021-00113-5

**Published:** 2021-05-10

**Authors:** Øyvind Sandbakk, Guro Strøm Solli, Rune Kjøsen Talsnes, Hans-Christer Holmberg

**Affiliations:** 1 Department of Neuromedicine and Movement Science, Centre for Elite Sports Research, Norwegian University of Science and Technology, Trondheim, Norway; 2 Department of Sports Science and Physical Education, Nord University, Bodø, Norway; 3 Meråker High School, Trøndelag County Council, Steinkjer, Norway; 4 Department of Health Sciences, Mid Sweden University, Östersund, Sweden; 5 Department of Physiology and Pharmacology, Karolinska Institutet, Stockholm, Sweden

**Keywords:** Altitude, Biathlon, Nordic combined, Cold temperatures, COVID-19, Jet lag

## Abstract

At the 2022 Winter Olympics in Beijing, the XC skiing, biathlon and nordic combined events will be held at altitudes of ~ 1700 m above sea level, possibly in cold environmental conditions and while requiring adjustment to several time zones. However, the ongoing COVID-19 pandemic may lead to sub-optimal preparations. The current commentary provides the following evidence-based recommendations for the Olympic preparations: make sure to have extensive experience of training (> 60 days annually) and competition at or above the altitude of competition (~ 1700 m), to optimize and individualize your strategies for acclimatization and competition. In preparing for the Olympics, 10–14 days at ~ 1700 m seems to optimize performance at this altitude effectively. An alternative strategy involves two–three weeks of training at > 2000 m, followed by 7–10 days of tapering off at ~ 1700 m. During each of the last 3 or 4 days prior to departure, shift your sleeping and eating schedule by 0.5–1 h towards the time zone in Beijing. In addition, we recommend that you arrive in Beijing one day earlier for each hour change in time zone, followed by appropriate timing of exposure to daylight, meals, social contacts, and naps, in combination with a gradual increase in training load. Optimize your own individual procedures for warming-up, as well as for maintaining body temperature during the period between the warm-up and competition, effective treatment of asthma (if necessary) and pacing at  ~ 1700 m with cold ambient temperatures. Although we hope that these recommendations will be helpful in preparing for the Beijing Olympics in 2022, there is a clear need for more solid evidence gained through new sophisticated experiments and observational studies.

## Introduction

Cross-country (XC) skiing, both on its own and as a key component of the biathlon and nordic combined events, is a challenging winter endurance sport, with athletes from more than 60 countries participating in international championships. In connection with the 2022 Winter Olympics in Beijing, XC skiing will play a major role in determining who wins 27 gold medals, more than 25% of the total to be awarded.

The XC skiing component in these sports involves competitions with race times ranging from multiple ~ 3-min races in sprint XC skiing (i.e., a qualification time-trial and three knock-out heats) to prolonged endurance distances of 30 km (women) and 50 km (men) lasting 1.5–2 h [55]. All these events are performed on varying terrain employing different sub-techniques of the classical and/or skating styles that involve upper- and lower-body work to different extents [27, 61, 62]. Consequently, elite performance requires mastering many different sub-techniques, as well as the ability to transition between these efficiently at speeds ranging from 5 to 70 km/h on inclines ranging from − 20% to + 20% gradients. Here, approximately 50% of the total time is spent racing uphill which is, therefore, the most important determinant of performance in time-trial races [3, 38, 48, 56]. However, 21 of the 27 competitions in Olympic XC skiing, biathlon and nordic combined events involve mass-starts or pursuits, in which tactics and sprint ability play additional roles in determining the final result.

The outdoor competitions during these winter-sports include alternations of environmental factors (i.e., altitude, weather and temperature), which requires physiological profiles and equipment adapted optimally to the prevailing environmental conditions. Accordingly, speed, work rate and energetics during XC skiing events must be adapted to the track profile, snow conditions, and the competition altitudes ranging from 0 to 1800 m [34, 55]. In addition to the demands of XC skiing, biathletes must simultaneously optimize shooting performance [31] and for nordic combined athletes, explosiveness and ski-jump performance are of importance [49, 69].

At the upcoming 2022 Beijing Olympic Games, the demands of XC skiing, biathlon and nordic combined events must be optimized for competing at an altitude of approximately ~ 1700 m, possibly under cold and dry environmental conditions and, for most of the athletes travelling east from world-cup competitions in Europe, following adjustment to the new time zone. In addition, the ongoing COVID-19 pandemic will likely limit the athletes’ ability to prepare optimally by modifying race programs and minimizing the involvement of support-teams. Accordingly, coaches and athletes must design effective preparation strategies to meet the complex and multidisciplinary demands under these challenging conditions and be prepared for sudden changes due to COVID-19.

Based on these new challenges, the current commentary provides evidence-based recommendations for the preparation for the Olympic XC skiing, biathlon and nordic combined events, as well as highlighting new areas to be explored in future research. Specifically, we discuss how to deal with elevated altitude, a cold environment, and eastern travel, as well as the possible impact of COVID-19.

## Endurance Demands Associated with XC Skiing, the Biathlon and Nordic Combined

Average speeds in XC skiing, biathlon and nordic combined races have almost doubled during the last 50 years, a trend that was accelerated by the introduction of the skating style in the middle of the 1980s. In addition, there have been a number of major changes in equipment and racing formats, with sprints, mass-starts and pursuits now being most common [31, 48, 55].

**Fig. 1:**
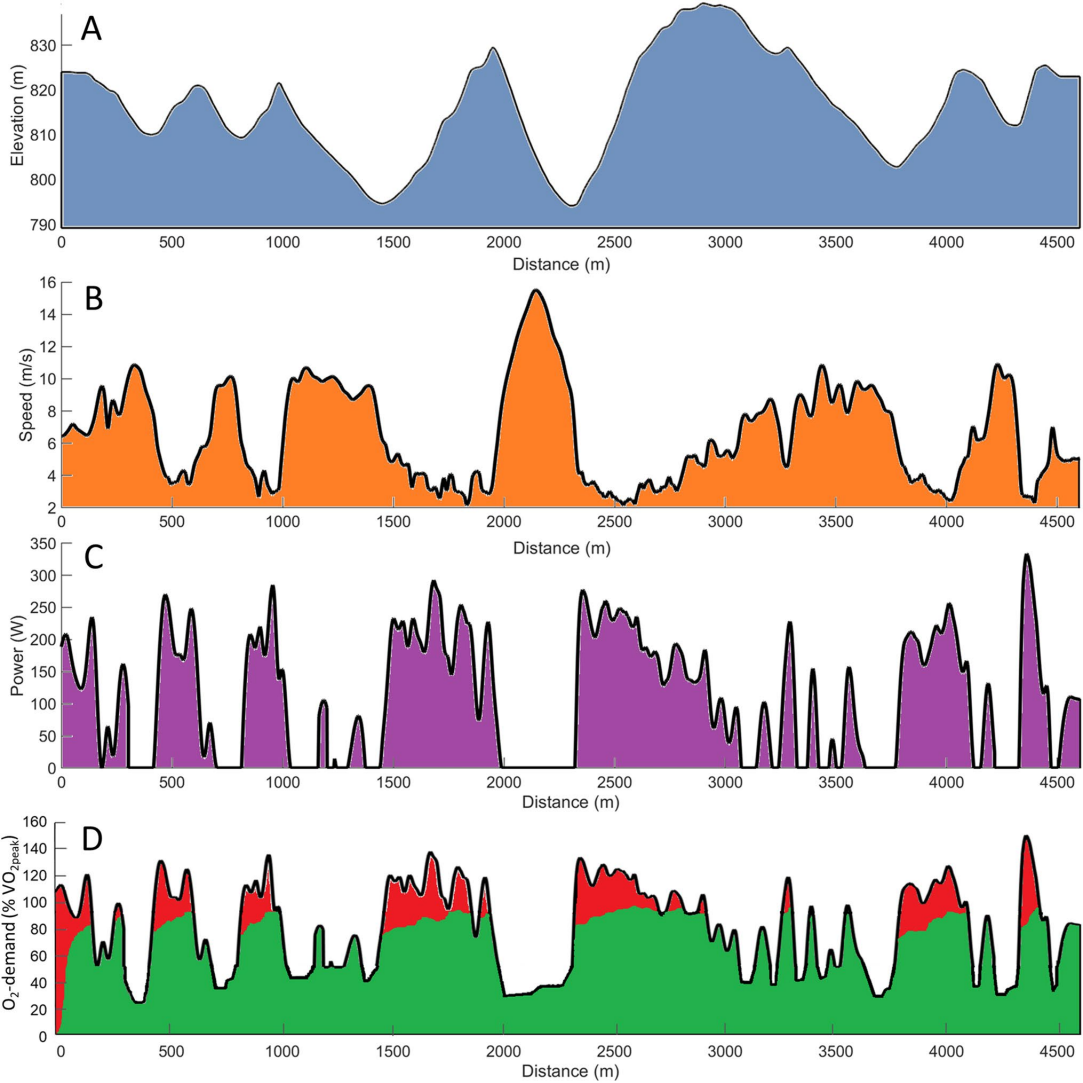
Variations in **a** elevation during a 5-km XC skiing race and associated changes in **b** speed, **c** estimated power output and **d** oxygen demand (green: aerobic contribution, red: anaerobic contribution). Note that these data are based on a single elite male cross-country skier during a competition with the skating technique, where the elevation profile and speed were measured with a highly accurate global navigation satellite system. Power was simulated on the basis of additional data on the body mass of this skier and his equipment, as well as estimated friction and drag coefficients (where the exposed area of the skier was estimated to change in connection with the use of the different sub-techniques at various speeds). To calculate the oxygen demand, we used the theoretical relationship between power and energy expenditure reported previously (for References, see [34, 55])

As mentioned above, speed during XC skiing competitions must be adapted to the track profile, snow conditions and altitude. The interval-based changes in speed and work rate across undulating terrain in XC skiing is referred to as micro-pacing, which creates a race format with increased work rate uphill and the possibility to recover downhill (Fig. 1) [25, 44]. On a macro-level, XC skiers and nordic combined athletes tend to use a positive pacing strategy, where the speed on any given type of terrain is lowered gradually as the race proceeds [48, 55, 66]. In contrast, biathletes seem to utilize a curvilinear speed-profile, with the fastest speed on the first and last lap [36, 37]. In all these events, the most successful skiers maintain speed better than their more poorly performing counterparts. Since these aspects are currently mostly studied during individual time-trial races, fewer details are known about mass-start races, in which the importance of drafting behind other skiers and obtaining a position that allows optimal utilization of the athlete’s individual strengths are accentuated.

On average, the aerobic contribution to total energy expenditure during XC skiing, biathlon and nordic combined competitions (i.e., 70%–75% during XC sprints and 85%–95% over longer distances) is comparable to the corresponding values for other sports, such as middle- and long-distance running, during similar racing times [21, 28, 34, 55]. Accordingly, the main performance-determining physiological capacities in XC skiing are maximal oxygen uptake (VO_2max_)**,** as well as the ability to reach a high peak oxygen uptake (VO_2peak_) and ski efficiently with the different sub-techniques [55]. However, the requirements for higher intensity on uphill terrain push the metabolic demands considerably above those required to elicit VO_2max/peak_, resulting in energy requirements as high as 40% and 15–20% above aerobic energy capacity in connection with sprint and distance races, respectively (Fig. 1) [34]. Hence, XC skiing also requires adequate anaerobic capacity and the ability to recover and reproduce anaerobic power [29, 35] during competitions. This might be particularly important in the less-studied mass-start events, and especially in biathlon and nordic combined events, where the uphill sections are normally shorter than for XC skiing. However, biathletes must simultaneously adjust their pacing strategy to optimize shooting performance, which may lead to a more conservative distribution of metabolic energy than in XC skiing and nordic combined [31].

All of the considerations mentioned above will also be influenced by the reduced availability of oxygen at the elevated altitude. In addition, the environmental conditions, which are expected to be cold and dry, will not only elevate snow friction but also challenge skiers with asthma, especially during rapid starts and on sections of a race where the work rate is extremely high. Another potential challenge may be alterations in the format of competitions in an attempt to reduce the likelihood of being infected by COVID-19. This could result in the performance of time-trail races with fewer competitors only or, perhaps, modified versions of mass-starts, pursuits or sprint events.

## Altitude Acclimatization

The altitude of ~ 1700 m at the Beijing Olympic Games is higher than at most world cup skiing competitions. The partial pressures of nitrogen (79%) and oxygen (21%) in the atmosphere will be considerably lower than normal [7]. In addition, bad weather associated with a reduction in atmospheric pressure could reduce this partial pressure of oxygen even further. Accordingly, the arterial blood will be less saturated with oxygen (i.e., 5.5% reduction per 1000-m elevation in altitude during maximal exercise), leading to less oxygen delivery to working muscles and a consequent impairment of endurance performance [7, 73].

Indeed, VO_2max_ decreases linearly by an average of 6.3% (4.5%–7.5%) per 1000-m elevation in altitude [73]. However, large inter-individual variations in the degree to which VO_2max_ declines with increasing altitude have been found, with several studies demonstrating larger reductions among well-trained endurance athletes with a high VO_2max_ compared to athletes with lower endurance capacity [8]. This has been linked to the ability to maintain arterial oxygen saturation during maximal exercise, which is lower in athletes with a high VO_2max_ even at sea level [10, 13]. Also, within more heterogeneous groups of endurance athletes, some athletes are more limited than others when competing at altitude, which may be caused by factors such as differences in the hypoxic ventilatory response and oxygen kinetics during exercise [8].

Although we focus mainly on the XC skiing demands in this commentary, biathletes must simultaneously optimize their shooting performance, which is physiologically more demanding at altitude compared to sea level due to a more pronounced increase in ventilation and heart rate during training and competition [39]. In addition, the ~ 30 s spent on shooting in biathlon leads to a start-and-stop procedure that is physiological challenging [31], especially at elevated altitudes, where athletes may recover more slowly.

The combined stressors of training and hypoxia at elevated altitude place increased demands on the athletes’ training and recovery routines, posing a larger risk for illness, maladaptation, overreaching and/or overtraining [2, 43]. In addition, athletes often report reduced duration and quality of sleep when exposed to altitude [54], possibly due to respiratory events and periodic breathing during sleep [30]. Overall, practitioners should be aware of these potential stressors when prioritizing training and/or living at elevated altitude for several weeks.

Among the several short- and long-term physiological adaptations to altitude that occur [7, 12], increased production of red blood cells (erythrocytes) and a higher total mass of haemoglobin in response to alterations in the level of erythropoietin (EPO) have been studied most extensively. Indeed, this is considered the main adaption that explains the improvements in performance resulting from altitude acclimatization [9, 43].

However, this adaptation normally occurs following at least 2–3 weeks of exposure to elevated altitude [9, 43] and improvement in performance at elevated altitudes involves other physiological adaptations as well [7]. The most important of these are more rapid and deeper breaths, both when resting and exercising, the so-called hypoxic ventilatory response [7]. This hyperventilation leads to a compensatory increase in blood pH (respiratory alkalosis) that inhibits further increases in ventilation, but this alkalosis can be eliminated within 4–7 days through bicarbonate secretion by the kidneys. Thereafter, pulmonary ventilation can be enhanced even further due to reductions in blood pH, which allow chemoreceptor-mediated enhancement of respiration [7].

Altitude training is common among elite endurance athletes [43], with world-class XC skiers (most of whom live at or close to sea level) typically performing ~ 10%–20% of their annual training at low-to-moderate altitude [55, 63, 70]. This training is usually distributed over 2–4 relatively short periods (e.g., ~ 14–18 days) each year, with training at 1500–3000 m, while living at ~ 1800–2000 m [55, 63, 70]. At the same time, the extent to which altitude training may enhance performance at sea level is widely debated [41, 60]. However, the current commentary will primarily discuss training at altitude for optimal acclimatization and performance during competitions at low- to-moderate altitudes (e.g. ~ 1200–1800 m).

In this context, simulating elevated altitude (i.e., hypobaric or normobaric hypoxia) utilizing different live low-train high strategies [42] might offer an effective alternative to actually training at elevated altitude for athletes preparing for the Beijing Olympics, especially since the ongoing COVID-19 pandemic limits travel. This approach may involve intermittent or chronic hypoxia without or in combination with exercise (e.g., treadmill roller-skiing). Although we are not aware of any controlled comparisons of the effects of simulated versus actual altitude training, exposure to simulated altitude during the last weeks prior to the Olympics may also be beneficial [7]. However, some evidence indicates that acclimatization is achieved most optimally by living and training at both the altitude and location at which the actual competitions are to be held [11]. In addition, to allow training sessions at higher speed/intensity while living at elevated altitude, training at sea level can be simulated under hyperoxic conditions (i.e., live high-train low). At the same time, it should be noted that in certain countries the use of hypoxic/hyperoxic methods has been abandoned entirely.

Overall, we suggest that athletes should plan their altitude acclimation with respect to the following three questions:

1. How many days prior to the competitions should they spend at elevated altitude to achieve optimal acclimatization?
2. At what altitude (relative to the altitude at which the competition is held) should they live and train?
3. During acclimatization, how should they change the training and recovery routines that they usually employ at sea level?

The optimal duration of acclimatization depends on the altitude at which the competition is to be held, as well as each individual’s requirements and previous experience of training and competing at elevated altitudes. Schuler et al. [59] exposed 8 elite cyclists to 2340 m and found 12.8% and 25.8% reductions in VO_2max_ and performance (i.e., time to exhaustion at 80% of maximal power output at sea level) respectively, already on the first day. Subsequently, both VO_2max_ and performance improved gradually during the initial 14 days at elevated altitude (4.6% and 13.6% reductions compared to sea level, respectively), with only minor improvement thereafter. Therefore, these investigators concluded that at least 14 days of acclimatization to elevated altitude is needed. It should, however, be noted that this study involved a live high-train low approach and that acclimatization to lower altitudes has been examined less extensively, although it seems likely that this would take less time. Potentially, non-physiological adaptations (e.g., changes in pacing strategies) may be more important at lower altitudes. Despite the sparse scientific evidence presently available, we would recommend that athletes acclimatize at an altitude similar to that of the Olympic venue for at least 10 days and, optimally, two weeks prior to the first event, especially in the case of competitions involving multiple events [7, 12, 14].

The large inter-individual variations in acclimatization demand that each athlete takes his/her previous experience in this respect into consideration [8]. Even though longer periods of acclimatization are regarded as more optimal [7], logistical and practical considerations (e.g., participation in important prior competitions at sea level) often lead to acclimatization for only 4–7 days. An alternative strategy is to go from sea level to an elevated altitude immediately before the competition (often referred to as the “fly-in and fly-out” approach), with the aim of reducing the acute effects of altitude exposure (e.g., reductions in plasma volume and buffer capacity, as well as in the quantity and quality of sleep) [20]. However, this is considered to be less effective than acclimatization for 10–14 days [7, 12, 14] and, therefore, appears to be irrelevant in the case of the Beijing Olympics, with its multiple events over the course of two weeks.

Another important consideration in this connection is the well-being of the athletes, which may be compromised by spending too much time at the competition venue prior to the first event. Thus, an alternative strategy would be to begin acclimatization at another location, while still arriving early enough at the actual venue so that athletes have time to acquaint themselves with the courses and plan their technical and tactical strategies.

The next question concerns the optimal living altitude for acclimatization for the competitions in Beijing. Chapman et al. [11] had four groups of collegiate distance runners live at different altitudes (1780, 2085, 2454 and 2800 m) and tested their running performance at 1780 m on Days 5, 12, 19 and 26. In this case, athletes living at the competition altitude (1780 m) demonstrated the least pronounced reduction in performance relative to sea level.

Nonetheless, many athletes perform the initial acclimatization prior to competition at an altitude higher than the competition venue to maximize erythropoiesis, before a tapering-off period with more emphasis on speed/intensity at the altitude of competition. Researchers and practitioners express differing views on this, and more experimental evidence is needed. Moreover, the training speed/absolute workload during acclimatization to elevated altitude is of significance. During the initial period of acclimatization to higher altitudes, much of the training is at relatively low speeds. Therefore, during the tapering-off phase, it would be advisable to conduct one or more competition-specific sessions of training at a higher speed/intensity.

Optimization of acclimatization and performance at elevated altitudes depends on various factors that affect training and recovery (i.e., training load, sleep, recovery and nutrition) [43]. The importance of nutrition for successful acclimatization to elevated altitude with the maintenance of good health is becoming more and more evident (for a full review, see Ref. [65]. Although more evidence-based recommendations are required for low-to-moderate altitudes, it appears that special awareness of enhanced utilization of carbohydrates and requirement for protein is warranted [65]. Furthermore, the increased ventilation at elevated altitudes leads to greater loss of water, thereby posing a higher risk for dehydration [65].

Particular attention should also be paid to the availability of iron and energy, as well as potential illness, which exerts a crucial influence on the increases in red blood cell volume and haemoglobin mass induced by hypoxia [65]. Consequently, ensuring adequate stores of iron (ferritin levels > 30 ng/mL) prior to exposure to low-to-moderately elevated altitude, together with individualized iron supplementation (~ 100–200 mg daily in oral form) during such exposure are to be recommended [2, 22, 65]. The risk of impairment in adaptations due to illness and/or injury related to systemic inflammation [65] can be minimized by appropriate preparation, periodization, programming and detailed monitoring of the athlete's training load and stress level during altitude acclimatization [68].

In addition to being acclimatized physiologically to the competition altitude, athletes must have gained the necessary competition experience through a sufficient number of competitions and training sessions at relevant altitudes in the years preceding the Beijing Olympics. Here, optimizing warm-up procedures and pacing strategies, as well as technique and tactics, are important. For example, the choice of pacing strategy may be particularly important with the lower availability of oxygen (and thereby risk for more oxygen debt early in the competition) at elevated altitude. Systematic use of technological developments, such as detailed training and competition analyses using high-accuracy global navigation satellite system (GNSS), should help athletes to understand (“calibrate”) the relationship between their perceived and actual intensity of exercise.

However, other aspects other than those mentioned above must also be considered when planning for the biathlon and nordic combined events. In nordic combined, the possibility to perform enough ski-jump specific training should be taken into consideration, especially at relevant altitudes. However, ski-jumping hills at ~ 1700 m are extremely rare, challenging nordic combined athletes and ski jumpers even more during these games. In the case of the biathlon, both mental and physiological factors for optimizing shooting performance must be considered as well.

Altogether, the experience of both training and competing at elevated altitude will be essential for optimizing the performance of endurance athletes during the Beijing Olympics. Important factors influencing training and recovery, as well as the actual optimization of performance must be developed in an environment of reduced oxygen availability. Here, athletes who normally live at elevated altitudes, as well as athletes who are highly experienced with training and competing at elevated altitude may have an advantage. Experience indicates that athletes may adapt more rapidly physiologically and gain more experience in optimizing training and performance at elevated altitudes by repeated sojourns at these altitudes. For example, during the years spent preceding the Salt Lake City Olympics in 2002, the most successful Scandinavian athletes trained at elevated altitudes for a total of 60–100 days on 4 or 5 different occasions.

**Fig. 2:**
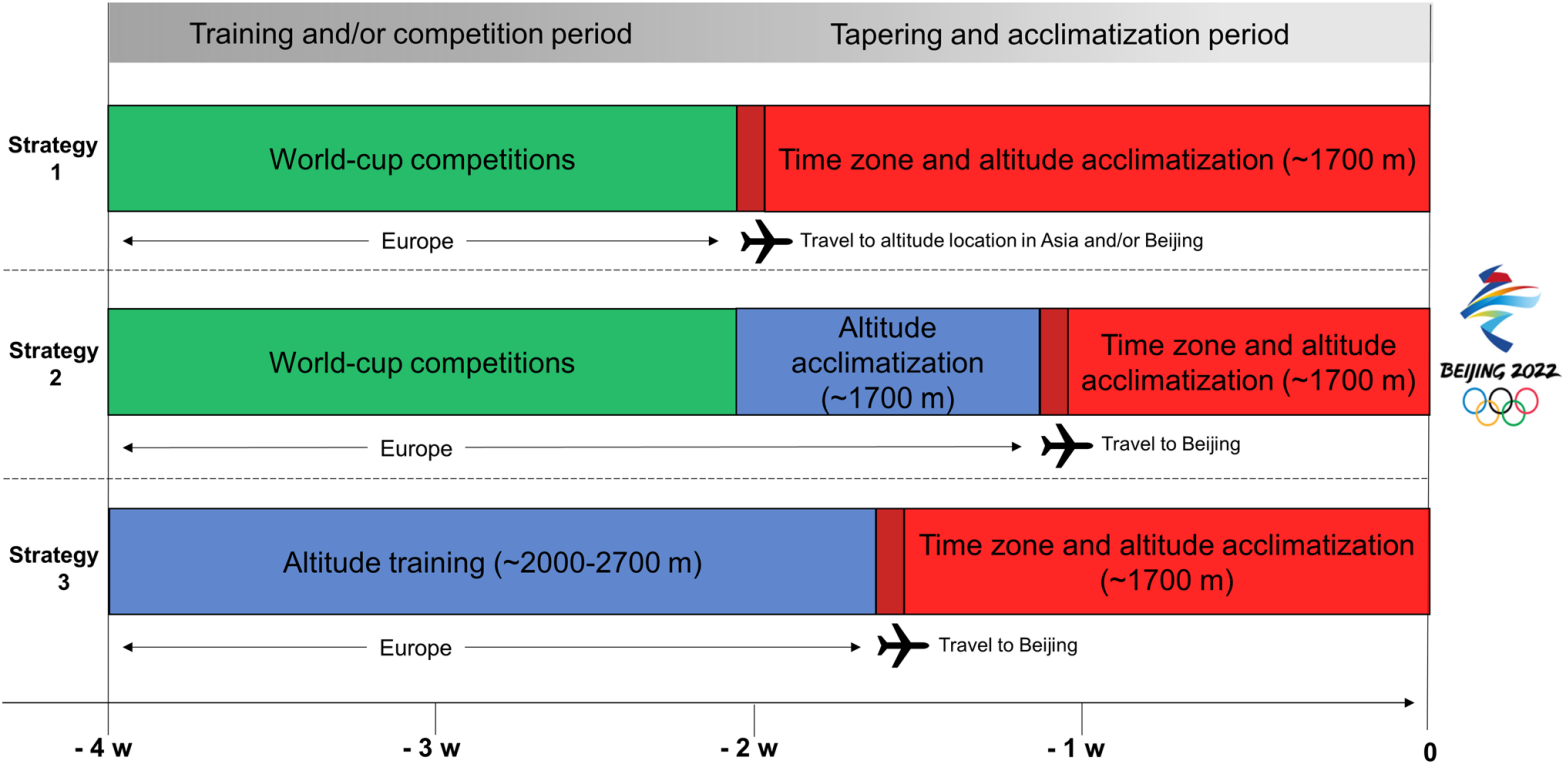
Illustration of three alternative strategies for the final period of preparation for the Beijing Winter Olympic Games in 2022

Our recommendations for optimal acclimatization to the elevated altitude of the Beijing Olympic games are listed in Table 1, while alternative strategies for the final preparations are suggested in Fig. 2.

**Table 1:** Major recommendations for optimizing preparations for the demands associated with the elevated altitude and cold temperatures at which the Beijing Olympics 2022 will take place

**Optimizing altitude acclimatization**	
**Long-term strategies**	**Short-term strategies**
During the year(s) prior to the Olympics, train for more than 60 days and participate in several competitions) at or above ~ 1700 m Learn how to adapt training and recovery routines, as well as pacing and technical strategies to elevated altitude Based on this training, develop individualized strategies for acclimatization, training, competition, recovery and nutrition in Beijing	10–14 days of acclimatization at an altitude similar to that at which the Olympic competitions will take place (**~ **1700 m) appears to optimize performance most effectively. This can be performed either at the Olympic venue itself or at other locations in Asia or in Europe (which may require later adjustment to the time zone in Beijing) An alternative strategy involves living and training for 14–21 days in a camp above 2000 m in order to promote erythropoiesis, followed by 7–10 days tapering off at ~ 1700 m. To maximize erythropoiesis, maintenance of adequate levels of iron (> 30 ng/mL ferritin) prior to altitude, in combination with individualized oral supplementation with iron (~ 100–200 mg daily) during altitude exposure is recommended
**Reducing travel fatigue and jet lag**	
**Before and during travel**	**After arrival**
Arrange to arrive at least one day earlier for each hour of difference between your time zone and that in Beijing During each of the 3 or 4 days prior to departure, shift your sleep and eating schedule by 0.5–1 h towards what it will be in Beijing To facilitate good sleep, take afternoon flights travelling east To avoid dehydration during travel, drink adequate volumes of non-alcoholic, non-caffeinated fluids	Plan timing of exposure to (day)light to optimize adaptation of your circadian rhythm and consider supplementation with 2–5 mg melatonin at appropriate times if needed Schedule meals and social contacts in accordance with the local time in Beijing Short (20–30 min) naps can aid recovery and help maintain normal alertness Avoid intensive training during the first few days after arrival
**Optimizing performance at cold temperatures**	
**Long-term strategies**	**Short-term strategies**
Optimize individualized warm-up procedures and pacing strategies at different temperatures Prepare clothing that is appropriate for different and potentially changing environmental conditions	Prepare clothing that ensure optimal maintenance of core temperature Develop a procedure for maintaining body temperature during the period between warm-up and the competition itself Adapt warm-up procedures and pacing strategies to ambient environmental conditions, with particular focus on the initial part of the race Athletes with asthmatic symptoms should plan for appropriate treatment in connection with high ventilation at cold temperatures

## XC Skiing in Cold Temperatures

Competitive XC skiers are typically exposed to ambient temperatures ranging from – 20 °C to as high as + 10 °C. The International Ski Federation allows competitions to be held at temperatures as low as − 20 °C [17] and the likelihood of low ambient temperatures during the Beijing Olympics is unusually high. With ambient temperatures of − 15 °C or lower the core body temperature rises, while the temperature of the skin falls due to cooling of the superficial musculature [32], which may subsequently influence its capacity to produce power and thereby reduce exercise economy [16, 46, 74]. In addition, previous reports indicate that wind may amplify these effects of cold temperature. These potential negative effects are associated with increased strain on the muscles and more rapid fatigue due to reduced oxidative enzyme activity, nerve and muscle excitability and nerve conduction [46]. In this respect, smaller athletes, with their relatively greater surface area and a lower amount of heat-producing muscle, might be influenced by cold weather to a greater extent than larger individuals.

Although endurance performance and work economy diminish as the ambient temperature falls, the effect on VO_2max_ is not as clear, with some reports documenting no effects and others a decrease in this parameter [1, 47, 57, 74]. Whereas some studies have reported no effects of ambient temperature on VO_2max_ [18, 58], others have indicated reduced VO_2max_ in cold conditions [47]. Still, relatively little is presently known about the effects of cold temperatures on endurance performance and related physiological mechanisms in connection with exercise that involves the entire body, such as XC skiing competitions, during which large volumes of cold air may be inspired.

Furthermore, competitions performed at altitudes of ~ 1700 m will also be associated with low humidity and therefore further challenge the ventilatory system, particular for asthmatic athletes. Accordingly, an athlete’s pacing strategy may change under cold conditions, since the role of pacing is to prevent unreasonably large homeostatic disturbances during the competition and to optimize performance [15, 74]. A particular challenge will be encountered by biathlon athletes, because of the stops for shooting, where fine motor skills are required for high precision.

Such challenges posed by cold ambient temperatures require the development of individually optimized warm-up procedures, and the strategies for keeping warm between the warm-up and start of the competition must be solved as optimally as possible. In addition, athletes must adapt their clothing to assure optimal thermodynamics both during warm-up and competition (e.g., optimization of race suits for the prevailing conditions). In addition, under these conditions asthmatic athletes must be allowed to develop medical treatment designed to prevent their airways from becoming limited and thereby to compete on an equal level with athletes without such problems. During the competition at cold temperatures, it seems likely that somewhat more conservative pacing strategies, especially in the initial part of the races, may be beneficial to prevent reduced performance at the end of the race [74]. However, our recommendations concerning how to handle cold ambient temperatures at the elevated altitude of the Beijing Olympic Games (Table 1) is not fully evidence-based and future studies are required to verify their effectiveness and possibly refine the strategies proposed.

In addition, changes in temperature affect the structure of snow, with consequent effects on the friction coefficient between ski and snow [4]. In contrast to most other endurance sports, XC skiers must adapt their physiological and technical abilities to the prevailing snow conditions of the day of competition. The combination of moderate altitudes and cold ambient temperatures at the Beijing Olympic Games will probably reduce the competition speeds [64], which may be beneficial for skiers with relatively low body mass and well-developed sub-techniques normally utilized at slower speeds. The possible abandonment of fluorine-based waxes may also lower speeds, although waxing teams are currently working systematically to find equally effective replacements.

## Travel Fatigue and Jet Lag

The world cup competitions in XC skiing, biathlon and nordic combined preceding the Beijing Olympics will be held in Europe. For athletes competing in these events, travel to Beijing involves crossing eight time-zones during 12–15 h flights, resulting in both travel fatigue and jet lag [71, 72]. Although many of the Nordic skiing competitions will be performed in the afternoon, which facilitates this adaptation, it will still be a major challenge. Both research on and the experience of the best athletes indicate that the physical and mental effects of jet lag can impair performance significantly [19, 71], and optimal performance at the Beijing Olympic Games, therefore, requires recovery and adjustment to the new time zone as quickly as possible. Athletes using altitude training in Europe as part of their preparation strategy may face particular challenges in combining altitude-induced effects with adjustment to a new time zone. Both the timing of travel to China and the quality of pre-camp locations may be crucial, and it is important for both the athletes and support team to understand the effects of travel fatigue and jet lag to effectively plan and individualize coping strategies (Fig. 2).

Jet lag involves a lack of synchronization between the established rhythm of biological functions (e.g., body temperature, heart rate, blood pressure, resting metabolism, intestinal function, brain activity, and hormone production) and the local time [53]. Although individual differences have been reported, the most common symptoms include poor night-time sleep, tiredness during the day, loss of appetite, gastrointestinal disturbances and impaired mental and/or physical performance [19, 40, 71, 72]. However, the circadian rhythm can be adjusted, and since this rhythm is somewhat longer than 24 h, it is easier to adjust backward (travel westward) than forwards (travel eastward, which most athletes will be doing for the Beijing Olympics). When traveling eastwards, a rate of adaptation of approximately one day for each hour of change in time zone has been proposed [19]. Accordingly, travelling from Europe to Beijing requires approximately 6–7 days of adjustment to the new time zone.

The hormone melatonin, the production of which is inhibited by light, is involved in regulating the circadian rhythm. Therefore, the adjustment of the circadian rhythm may be facilitated by appropriately timed light exposure/avoidance and/or ingesting melatonin, as well as by exercise [19, 53, 71]. Regarding light exposure, natural daylight seems to be more effective than artificial light [50]. Exogenously administered melatonin can also be used to accelerate the shift in the circadian rhythm. A meta-analysis reported that intake of 0.5–5 mg melatonin close to the target bedtime at the destination decreased jet-lag from flights crossing > 4 time zones [26]. Timing of light exposure and melatonin can be used independently, but using them together seems to produce a more significant effect [6, 76]. However, more evidence-based recommendations on the effects of exogenous melatonin on reducing jet lag and travel fatigue in athletes crossing multiple time zones are required.

### Before Departure to Beijing

Because most athletes perform best in the late afternoon (approx. 4–6 PM), when the body temperature is highest [52, 75], athletes traveling east will often perform worse in a competition that takes place early in the day at the new destination. Accordingly, the timing of the competitions during the Beijing Olympic Games can potentially also affect performance. To prepare for this, athletes could perform key sessions (e.g., high-intensity training) or competition in their home country at a time which corresponds to the time of day when their competition is scheduled.

Sleep is an essential aspect of recovery and performance in athletes and sleep deprivation has been associated with increased errors, impaired decision making, reduced maximal power, and more pronounced fatigue, as well as reduced ability to perform the maximal exercise and increased risk of illness [23, 45, 51]. To minimize the disturbance of sleep during preparation for the Beijing Olympics, it may be beneficial to start adjusting one’s sleep pattern 3–4 days before departure by shifting the sleep schedule by one hour each day [33]. Large adjustments in bedtime and wake-up time can, however, work against their purpose and disrupt the circadian rhythm, becoming socially disruptive with potentially adverse effects on the training performed during the days before departure [53].

### While Travelling

We recommend that athletes book afternoon flights to facilitate sleep as much as possible when travelling. However, long flights, regardless of time zones, can also result in discomfort and fatigue due to cramped seats, changes in meal rhythms, low air quality, limited sleep opportunities and reduced mobility [72]. Such conditions can aggravate the symptoms of jet lag, as well as impair acclimatization and recovery following a long journey. Travelling poses a larger risk of illness, e.g., Svendsen et al. [68] reported that international air travel is the single most important risk factor for upper-respiratory tract and gastrointestinal infections in elite XC skiers. In addition to respiratory symptoms, gastrointestinal sickness related to traveling is common among athletes [24]. Thus, careful planning and organization are required to minimize the risk of infection during the long air travel to Beijing. Avoiding contact with commercial travellers by chartering a plane may be a beneficial strategy for nations with many athletes traveling together.

Other risks associated with frequent travel include inadequate nutrient intake and dehydration [24]. Athletes should attempt to minimize the dehydrating effects of flight by drinking adequate amounts of non-alcoholic fluids. Caffeine should be avoided as this can act as a diuretic and also interfere with the timing of sleep designed to adjust effectively to a new time zone. The use of compression socks should be considered to improve the venous return of blood from the legs [5]. After departure, practitioners should set the clock as soon as possible to Beijing time [72].

### After Arrival in Beijing

To advance the circadian rhythm when traveling eastwards, it may be beneficial to avoid exposure to (sun)light before midday (local time) and maximize exposure to sunlight after midday [52, 53]. Therefore, if the plane lands early in the morning, the use of light screens on the plane and wearing dark glasses (possibly best are those that block out blue light) both at the airport and on the way to the hotel might be a good alternative. In this way, athletes can sleep for a few hours on arrival [51]. In the days after arrival exposure to light can be shifted gradually 1.5 h earlier each day to advance the circadian rhythm towards Beijing time [53].

Exercise has a likely positive impact on the adjustment of the circadian rhythm. However, the athletes’ training sessions should be timed in accordance with the recommendations for light exposure [71]. Also, an exercise in the morning should be avoided for a few days after traveling east, as this can prolong adjustment of the circadian rhythm [51], and strenuous training sessions should be avoided during the first few days [19]. However, further research on the optimal quantity and type of exercise for appropriate alteration of the circadian rhythm is required [19, 53].

Finally, nutrition may have an important role in the adjustment of the circadian rhythm. Meals high in carbohydrates and low in protein for the evening and a high-protein breakfast have been suggested to facilitate adaptation to the time at the destination [33]. However, the effect of such specific dietary interventions appears to be uncertain and the timing of meals to adapt to the new environment seems to be more important than the distribution of nutrients [19, 71, 72].

Although we present here existing guidelines for optimal adjustment to changes in time zone, it is important to be aware that the scientific evidence on how athletes can best recover from travel fatigue and jet lag is relatively limited. The large intra-study variation in the determination of circadian phase complicates previous findings, as does the wide variety of outcome measures [71]. In addition, the simultaneous acclimatization to altitude complicates the picture.

Again, our recommendations for reducing fatigue and jet lag when traveling to the Beijing 2022 Olympics are listed in Table 1 and alternative strategies for the final preparation period suggested in Fig. 2.

## COVID-19

The global COVID-19 pandemic has changed our world dramatically in many ways, including limiting the possibilities for athletes to train and compete. Currently, we do not know how the Beijing Olympic Games will be affected. Consequently, while athletes and coaches must prepare as optimally as possible, they must also keep in mind the many restrictions that may be placed on preparations and competitions in the future. Indeed, alternative plans for solving the challenges associated with acclimatization to altitude and changes in time zone, as well as preparing for competition in cold environments must be prepared. However, the rapid development and deployment of COVID-19 vaccines will hopefully attenuate the global pandemic, allowing the 2022 Olympic Games to be held in a reasonably normal fashion and athletes to prepare optimally.

Some of the main changes and restrictions that might occur are listed below together with possible solutions to the problems:

- Competitions with a reduced number of competitors, reduced support-teams and without spectators must be expected. This will challenge athletes to take more responsibility for their training, equipment and waxing, as well as recovery and mental preparations (e.g., attaining their full potential in the absence of spectators), while coaches and support-teams must prepare to work more efficiently and organized than previously.
- Other types of competitions may be held, with a likelihood of more individual time-trial races that require different and more individualized pacing strategies. New technology might help skiers to train for this, and by using GNSS technology combined with video, the competition tracks can be simulated during training both outdoors and virtually during roller skiing on treadmills. On treadmills, this can even be done at simulated altitude in nations where such strategies are allowed.
- Cancelled or restricted world cup competitions in the preparations for the Olympics will limit the possibilities both for qualifying for the Olympics and for optimal preparation for competition. Accordingly, each nation must have alternative strategies for handling these challenges as well as possible.
- Routine testing for COVID-19 and possible quarantine in connection with competitions could also limit training and preparation. Indeed, the current rules in China might not allow many athletes to be outdoors, but whether this is realistic or will inevitably lead to the cancellation of the Olympics remains to be seen.

In addition, the considerable reduction in testing for doping due to the pandemic will lead to increased problems of this nature, both in connection with preparation for and during the Olympics. For example, in the endurance events, manipulation of the volume of erythrocytes in the blood to improve transport of oxygen may become more common. Half of the medallists at the World Championship in Lahti 2001 were found to have highly abnormal haematological profiles [67] and many skiers who had won medals have also been found guilty of blood doping during the past two decades. Although it appears that in general mean haemoglobin levels in elite athletes have been falling due to better anti-doping programs, including more tests, better test procedures and the introduction of blood passes, this type of control is now more limited. Thus, to allow fair competition, international federations and the Olympic organizers must strive to improve this situation.

## Conclusions and Future Research Perspectives

In addition to the intrinsic demands associated with XC skiing, the biathlon and nordic combined events, the integrated implications of low-to-moderate altitude, cold climate, and eastern travel across multiple time-zones, as well as the possible limitations due to the COVID-19 pandemic motivate us to formulate the following evidence-based recommendations for athletes preparing for the Beijing Winter Olympics 2022:

- Ensure extensive experience of training (> 60 days annually) and competition at or above the altitude of competition (~ 1700 m), to optimize and individualize your strategies for acclimatization and competition.
- In preparing for the Olympics, 10–14 days at ~ 1700 m seems to optimize performance at this altitude effectively. An alternative strategy involves two–three weeks of training at > 2000 m to maximize erythropoiesis, followed by 7–10-days of tapering off at ~ 1700 m.
- Optimize athletes' own individual procedures for warming-up, as well as for maintaining body temperature during the period between the warm-up and competition, effective treatment of asthma (if necessary), optimal clothing and pacing at ~ 1700 m with cold temperatures.
- During the last 3–4 days prior to departure, gradually shift your schedule for sleeping and eating by 0.5–1 h each day towards local time in Beijing, at the same time as you avoid inappropriate exposure to light. We recommend arriving one day earlier for each hour of time adjustment; exposure to daylight in a manner that facilitates adaptation of the circadian rhythm; timing your meals, social contacts, and naps in an appropriate manner; and initiate training in Beijing at a relatively low intensity, gradually increasing this intensity as the body becomes adjusted.
- The unknown way in which the COVID-19 pandemic may limit training and competition calls for coaches and athletes to make alternative plans to meet complex and multidisciplinary demands, always being prepared for sudden changes.

Although we hope that these recommendations will be helpful in preparing for the Beijing Winter Olympics in 2022, many of them are based on experience in combination with insufficient scientific evidence. There is a clear need for more research-based evidence, which could be obtained both through sophisticated experiments and observational studies employing new technology to simultaneously monitor indicators of performance, physiological responses and environmental factors in connection with altitude training, cold exposure and travel across time-zones.
